# Healthcare and cost burdens associated with potentially inappropriate medication use in older adults: a matched cohort study

**DOI:** 10.1186/s12877-026-08057-7

**Published:** 2026-07-28

**Authors:** Jaeok Lim, Sohyun Jeong, Arum Moon, Jung-Ha Kim, Sunmee Jang

**Affiliations:** 1 College of Pharmacy, Gachon University, 191 Hambakmoei-ro, Yeonsu-gu, Incheon, 21936 Republic of Korea; 2 School of Pharmacy, Massachusetts College of Pharmacy and Health Sciences, Boston, MA 02115 USA; 3 Gachon Institute of Pharmaceutical Sciences, Incheon, Korea; 4 Department of family medicine, College of Medicine, Chung-Ang University, 102 Heukseok-ro, Dongjak-gu, Seoul, 06973 Republic of Korea

**Keywords:** Potentially inappropriate medication, Hospitalization, Emergency department visits, Healthcare cost

## Abstract

**Background:**

The adverse effects of potentially inappropriate medications (PIMs) use in older adults are one of major public health burdens. This study aimed to examine the association between PIM use and healthcare utilizations and costs among older adults.

**Methods:**

We used the Korean National Health Insurance Service-Elderly Cohort Database in 2015–2018 with matched cohort design. A propensity score derived from 16 variables, including age, sex, index year, Charlson Comorbidity Index scores, income decile, residential status, nine main comorbidities and number of comedication, was used for 1:1 matching. Cox proportional hazards regression was used to compare the risk of hospitalization and emergency department (ED) visits 1 year after the index date between PIM users and non-users. In addition, hospitalization- and ED visit related costs were evaluated using a two part model consisting of logistic regression and generalized linear model with logit link.

**Results:**

A total of 3,896 older adults aged ≥ 65 years at index date were included, comprising 1,948 PIM users and 1,948 matched non-users. All the matching variables were well balanced with absolute Standardized Mean Difference (aSMD) < 0.1. Females were 52.77% in non-users and 54.65% in users. The mean age was 73.26 (sd: 5.97) in non-users and 73.14 (sd: 5.98) in users. The results indicated that PIM users were associated with higher risk of hospitalization than non-users (adjusted HR [aHR]: 2.18, 95% CI: 1.81–2.63) and were more likely to visit the ED (aHR: 3.08, 95% CI: 2.01–4.72). Furthermore, the total healthcare costs for PIM users were 40% higher among PIM users than non-users (adjusted Cost Ratio [aCR]: 1.4, 95% CI: 1.35–1.54). Among PIM users, increasing numbers of PIMs were associated with higher total healthcare costs, as well as higher hospitalization- and outpatient-related costs.

**Conclusion:**

PIM use was associated with an increased risk of hospitalization and ED visits, as well as higher healthcare costs. These findings highlight the importance of regular medication review and optimization of prescribing in older adults. However, PIM use should be considered within the broader context of multimorbidity and frailty, rather than as an isolated risk factor, when developing strategies to improve health outcomes in this population.

**Supplementary Information:**

The online version contains supplementary material available at https://doi.org/10.1186/s12877-026-08057-7.

## Background

Older adults have the increased risk of polypharmacy and drug related problems due to their multiple common conditions [1, 2]. Especially, adverse effects to older adults caused by potentially inappropriate medications (PIMs) are one of major public health concerns. PIMs can be defined as medications in which the risk of adverse events outweighs the clinical benefits where there is a safer and more effective alternative [3].

Globally, PIMs had a pooled prevalence of 36.7%, and PIMs use among older patients has become increasingly prevalent in the past 2 decades according to the recent meta-analysis [4]. This study also found that the pattern and burdens of PIM use were different by geographic environment and economic income differences. Africa showed the highest prevalence (47.0%) followed by South America (46.9%), and the lowest in Oceania (23.6%) [4]. A US study estimated that from 2014 to 2018, 43 billion doses of PIMs were dispensed to the Medicare Part D population, with spending on PIMs of $25.2 billion [5]. The most common PIMs in older adults include proton pump inhibitors (PPIs), benzodiazepines, and tricyclic antidepressants [5].

PIMs have been associated with hip fractures [6], cardiovascular events [7], hospitalizations [8, 9], emergency department visits [9] and overall increased public health services utilization [10]. There were mixed results for the association between PIMs and mortality [7, 11]. The use of PIMs by older adults increased medical resources and healthcare costs but evidence on costs was reported inconsistent [12, 13].

Thus, this study aimed to examine the association between PIM use and adverse health outcomes as well as to quantify the overall healthcare burdens and cost associated with PIM use using a nationwide cohort.

## Methods

This study uses the similar approach as our previous self-controlled case series (SCCS) study on the use of PIMs in older adults regarding sample dataset (NHIS-ECDB), definitions over PIMs, comorbidities and part of outcomes of interest [9]. However, the analytical dataset, study population, study design, and statistical methods differ substantially between the two studies. Our previous study constructed an SCCS dataset that included only individuals who experienced hospitalization or ED visits during the study period (case only design). In the present study we newly constructed a propensity score-matched cohort of PIM users and non-users from the same source database, the cost outcomes were evaluated, and different years of datasets were used. The detailed and key approaches of current study are outlined below.

## Database

Korean National Health Insurance Service-Elderly Cohort Database (NHIS-ECDB) was utilized (REQ202400978-014). NHIS platform established ECDB as a sample representative research database to enhance and support medical and pharmaceutical research activities in various sectors. The database provides insurance claim-related multiple information on individuals over the age of 60. They are age, sex, death, disability, income tertile, medical aid status, healthcare service utilization, long-term nursing home services, diagnosis, medical and dental treatment, prescription information and all cost information (National Health Insurance Sharing Service, Sample Research DB, https://nhiss.nhis.or.kr/bd/ab/ bdaba 022O eng. do#). This study used data from 520,251 individuals from January 1, 2015 to December 31, 2018.

### Study population

New PIM users were required to have at least 1 PIM prescription and had following inclusion criteria; [1] older adults who aged ≥ 65 y at index date (the date when the first PIM prescribed) [2], no PIM prescriptions 12 months before index date [3], no history of outcomes of interest (hospitalization or ED visits) in 12 months before index date, and [4] no cancer history and death during the entire study period, which is to prevent any potential confounding from highly frail individuals and complicated treatment regimens. PIM non-user was selected when they met the above-mentioned criteria as well as no history of PIM prescription during the follow-up period. Index date of PIM non-user was the first clinic visit date after 1 year of no PIM use period.

### Outcomes and exposure definition

The Beers Criteria are one of the most widely used explicit lists of PIMs for the older adults [3]. PIM drug lists were defined by the information of 2019 updated Beers criteria [14]. We assumed it is appropriate to apply 2019 BEER’s criteria since Korean NHIS-ECDB we used in this study had datasets up until the year of 2019 when we conducted this research. We acknowledge that healthcare policies and clinical guidelines may influence clinical practice, including prescribing patterns and medication selection [15] which could potentially lead to differences in study outcomes when different versions of the Beers Criteria are applied. The PIM categories are anticholinergics, antithrombotic, anti-infectives, cardiovascular medications, central nervous system medications, endocrine medications, gastrointestinal medications (PPIs and metoclopramide), pain medications (NSAIDS and muscle relaxant), and genitourinary tract medications. All PIMs used in this study are presented in Supplementary data (Table S1).

The outcomes of interest were hospitalization, ED visits and healthcare costs (excluding patient copays) associated with those events at 1 year after exposure. ED visits continued to hospitalization were considered as hospitalization event, whereas for cost evaluation, ED visits continued to hospitalization were separated for each ED visit and hospitalization cost calculation.

Healthcare costs were measured in terms of costs of medication, inpatient treatment, outpatient physician services, medical supplies, and rehabilitation. Out of pocket (uninsured) costs were not considered since NHIS-ECDB only contains insurance reimbursement information. All costs were summed up to obtain a variable that represented respective total costs per inpatient, outpatient and ED visits. The Korean Won is represented by the US. Dollar based on the exchange rate as of 12.31.2018 (1 US dollar = 1113.5 Korean Won).

The measurement of PIM exposure duration was calculated as the simple sum of the prescribed days for all PIM prescription claims occurring during the 1-year observation period. If the end date of a prescription extended beyond the individual’s 1-year observation period, the prescription end date was truncated to the last day of the observation period. Accordingly, gaps between prescription periods were not included in the PIM exposure duration. Overlapping prescription periods were counted multiple times, and the cumulative PIM exposure duration was truncated at 365 days if it exceeded 365 days.

### Statistical analysis, covariates and matching

Basic patient characteristics such as age and sex, information on comorbidity, socioeconomic status such as income decile and residential status in metropolitan areas were collected and presented.

1:1 Matching between PIM user and PIM non-user was performed using covariates derived propensity score. Matching variables were age group, Charlson Comorbidity Index (CCI) scores, income decile, residential area (metropolitan area or rural area), nine major comorbidities (cardiovascular disease, cerebrovascular disease, psychiatric disease, hyperlipidemia, diabetes, liver disease, COPD, osteoporosis, and fracture), and number of comedication. In this study, the number of PIM users (case) was much higher (*n* = 48,938) than PIM non-users (control, *n* = 16,929). We managed to have 9–10 times higher number of controls available to ensure a certain number of PIM users (case, we performed 4% random sampling from PIM users, that is 1,957) to fully match controls [16].

Comorbidity information was assessed using ICD-10 diagnosis codes in the previous 1-year of cohort enrollment (Table S2). Since psychiatric diseases were highly confidential in Korea, we used ICD-10 code (F.xx) to capture a broad psychiatric disease. We assumed 1 month of period is a reasonable period to capture patients’ medication status immediately before cohort entry rather than cumulative historical medication exposure and the number of co-medication was matched as well.

Variables not included in matching such as hypertension, atrial fibrillation, heart failure, chronic kidney disease, arthritis, and residential status in long-term care facility were adjusted in the statistical model afterwards. We estimated the risk of hospitalization and ED visits using a Cox proportional hazard regression model and presented the crude hazard ratio (cHR) and adjusted HR (aHR) after covariate adjustment in the model.

The baseline characteristics of the included older adults were represented by descriptive statistics by numbers and percentages in discrete variables or mean and standard deviation in continuous variables. In addition, mean differences of continuous variables were tested by t-test, discrete variables were tested using chi-square tests.

For the healthcare cost analysis, We used a two-part model because substantial proportion of cost had zero values when we included all the matched study participants regardless of having outcomes of interest. A two-part model handles data with a large true zeros and a continuous positive distribution (e.g., healthcare costs or insurance claims). It splits the data into two sequential equations: a binary model (Part 1) to predict the probability of a non-zero outcome, and a continuous model (Part 2) for the magnitude of positive values [17]. In the first part, logistic regression was used to model all the study participants including who did not have hospitalization or ED visits, where comparison is presented in Odds Ratio (OR). In the second part, generalized linear models (GLM) with a log link and gamma distribution were used to estimate healthcare costs among individuals who had hospitalization or ED visits and incurred costs associated with those outcomes. In the second part, we interpret the results as times of increased cost of PIM user compared to non-PIM user (cost ratio, CR) This approach was selected because the expenditure data tend to be right skewed best fitting to gamma distribution [18]. The natural logarithm of healthcare costs was additionally used to obtain normal distribution.

All analyses were performed using SAS version 9.4 and significance was determined with two-sided 95% CI and a *p* value < 0.05.

## Results

### Baseline characteristics

**Fig. 1:**
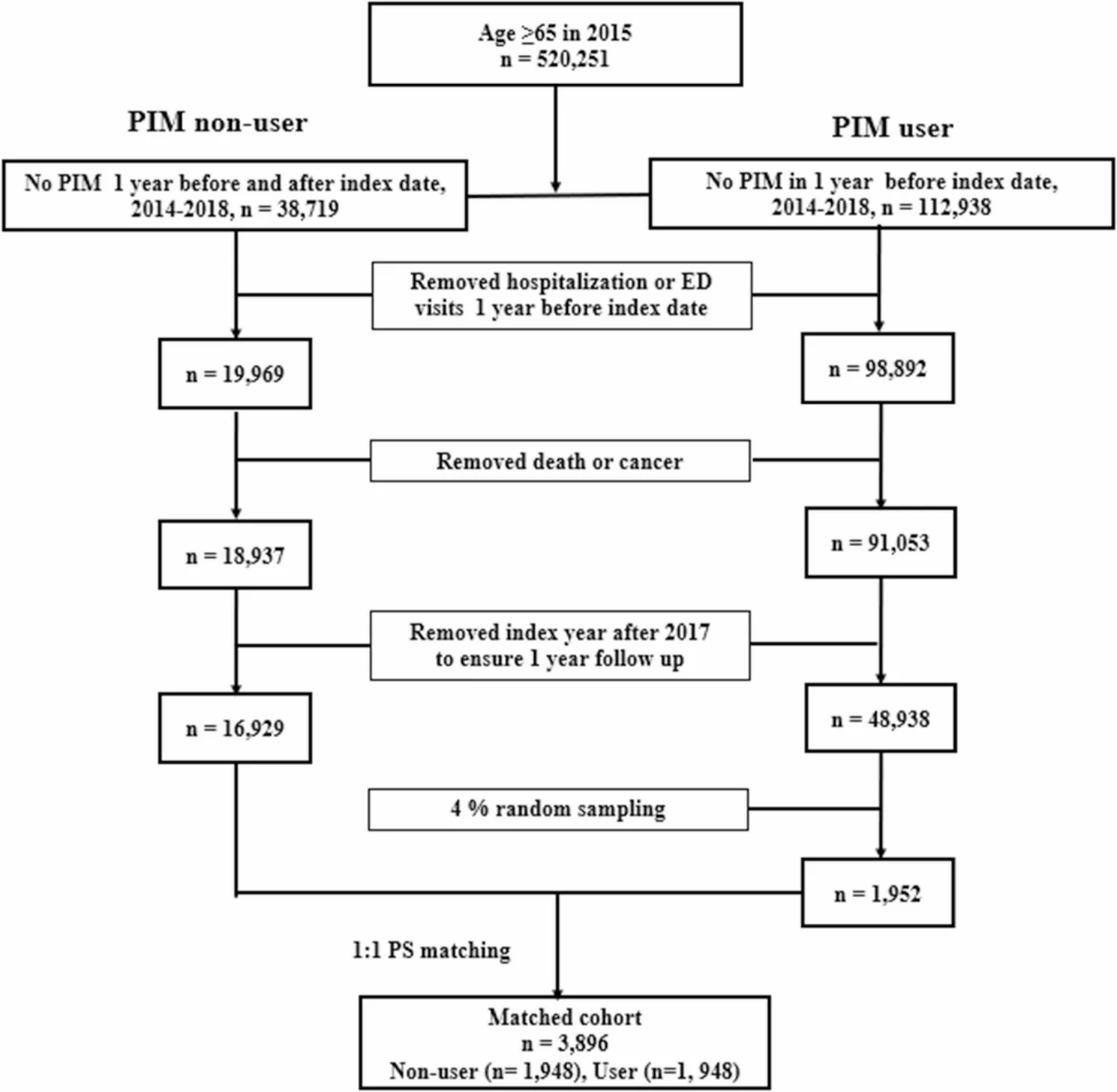
Selection flow of study participant. PIM: Potentially inappropriate medication, PS: Propensity score

After applying all inclusion and exclusion criteria, eligible individuals for PIM users were 48,938 and non-user 16,929. PIM users were 2.98 times higher than non-users during the period of 2015–2018. Owing to the higher number of PIM users, we selected 4% sample (*n* = 1,957) by algorithm generated random selection to make the matching process proper, since providing at least 10 times control individuals is appropriate for 1:1 matching. The final 1:1 matching was well matched with *n* = 1,948, a total of 3,896 for PIM non-user and PIM users. The whole selection flow is presented in (Fig. 1).

Before and after matching information with aSMD information is presented in Table S3. All the matching variables were well balanced with aSMD < 0.1 (Table S3). After matching, females were 52.77% (*n* = 1,028) in non-user and 54.67% (*n* = 1,065) in user. The mean age was 73.26 (sd: 5.97) in non-user and 73.14 (sd: 5.98) in user. Most individuals included in this study have CCI score 0 (75.21% in non-user and 73.97% in user), ischemic heart disease history (4.26% in non-user, 4.93% in user), diabetes (8.32% in non-user, 9.09% in user) and other comorbidities less than 6% in each group. The groups have less than 10 co-medication profiles (79.06% in non-user, 78.8% in user). The metropolitan area residence and income decile which might affect healthcare accessibility and financial status were well balanced as well. In the unmatched variables, hypertension was higher in non-user (47.74%) whereas it is 38.19% in user. Long-term care facility residents were higher in non-user (2.16%) versus in user (1.28%). The baseline characteristics are presented in (Table 1).

**Table 1:** Baseline characteristics of matched data

Variable	Category	PIM non-user (*n* = 1,948)	PIM user (*n* = 1,948)	aSMD	*p*-value
Matched covariates					
Year, n, (%)	2015	857 (43.99)	857 (43.99)	0	1
	2016	648 (33.26)	648 (33.26)		
	2017	443 (22.74)	443 (22.74)		
Sex, n (%)	Female	1028 (52.77)	1065 (54.67)	0.038	0.235
	Male	920 (47.23)	883 (45.33)		
Age, mean (sd)		73.26 (5.97)	73.14 (5.98)	0.02	0.527
Age group, n (%)	65–74	1193 (61.24)	1252 (64.27)	0.063	0.069
	75–84	657 (33.73)	590 (30.29)	0.074	
	85-	98 (5.03)	106 (5.44)	0.018	
CCI Score, n (%)	0	1465 (75.21)	1441 (73.97)	0.028	0.519
	1	373 (19.15)	395 (20.28)	0.028	
	2	78 (4)	71 (3.64)	0.019	
	3+	32 (1.64)	41 (2.1)	0.034	
Income decile, n (%)	0 (no income)	130 (6.67)	118 (6.06)	0.025	0.861
	1–4	474 (24.33)	475 (24.38)	0.001	
	5–8	643 (33.01)	639 (32.8)	0.004	
	9–10 (highest)	701 (35.99)	716 (36.76)	0.016	
Living in a metro city, n (%)		870 (44.66)	866 (44.46)		0.004
Comorbidities, n (%)	Ischemic heart disease	83 (4.26)	96 (4.93)	0.032	0.320
	Cerebrovascular disease	74 (3.8)	81 (4.16)	0.018	0.566
	Psychiatric disease	104 (5.34)	111 (5.7)	0.016	0.623
	Hyperlipidemia	89 (4.57)	97 (4.98)	0.019	0.548
	Diabetes	162 (8.32)	177 (9.09)	0.027	0.394
	Liver Disease	22 (1.13)	21 (1.08)	0.005	0.878
	COPD	81 (4.16)	106 (5.44)	0.06	0.061
	Osteoporosis	92 (4.72)	101 (5.18)	0.021	0.506
	Fracture	15 (0.77)	15 (0.77)	0	1.000
Number of comedication (mean, sd)		7 (3.29)	7.12 (3.26)		0.04
Group by number, n (%)	G1:1–5	717 (36.81)	682 (35.01)	0.038	0.291
	G2:6–9	823 (42.25)	853 (43.79)	0.031	
	G3:10–14	355 (18.22)	363 (18.63)	0.011	
	G4:15–19	51 (2.62)	43 (2.21)	0.027	
	G5:20-	2 (0.1)	7 (0.36)	0.054	
Unmatched variables					
Comorbidities, n (%)	Hypertension	930 (47.74)	744 (38.19)		< 0.01
	Atrial Fibrillation	38 (1.95)	22 (1.13)		0.037
	Heart Failure	37 (1.9)	20 (1.03)		0.023
	CKD	29 (1.49)	9 (0.46)		0.001
	Arthritis	177 (9.09)	240 (12.32)		0.001
Long-term care, n (%)	Facility resident	42 (2.16)	25 (1.28)		0.036
	Home resident	95 (4.88)	54 (2.77)		< 0.01

*PIM* Potentially Inappropriate Medication, *SD* Standard Deviation, *CCI* Charlson Comorbidity Index, *COPD* Chronic Obstruct Pulmonary Disease, *CKD* Chronic Kidney Disease, *aSMD* absolute Standardized Mean Difference

*Number of individuals having at least one hospitalization/ED visit at 1 year

#Consecutive hospitalization in the same institution having multiple claims were defined as one episode

Significant results made in bold type

### Outcomes

#### PIM use by PIM categories

PIMs were categorized as pain medications, anticholinergics, gastrointestinal medications, genitourinary medications, cardiovascular medications and endocrine medications. The highest prevalent medication was pain medications constituting 87.94% of individuals. Next was anticholinergics, 69.3% individuals used it followed by gastrointestinal medications which were used by 30.49% individuals. 63.34% individuals used less than five PIMs, mean number of PIMs used were 4.17 (sd:2.69). The mean duration of PIM prescription was 77.42 days (sd: 92.95). The highest proportion of individuals (23.25%) used PIMs for 31–60-day duration (Table 2).

**Table 2:** Information and distribution of PIMs used in PIM users

Variable	Category	PIM user (*n* = 1,948)
PIM, n (%)	Pain medications	1,713 (87.94)
	Anticholinergics	1,350 (69.3)
	Gastrointestinal medications	594 (30.49)
	Genitourinary medications	85 (4.36)
	Cardiovascular medications	40 (2.05)
	Endocrine medications	29 (1.49)
Number of PIM, mean (sd)		4.17 (2.69)
Group by number of medications, n (%)	G 1: 1–2	594 (30.49)
	G2: 3–4	640 (32.85)
	G3: 5–6	387 (19.87)
	G4: 7–18	327 (16.79)
Duration of PIM, mean (sd)		77.42 (92.95)
Group by duration of medications, n (%)	1–15 days	411 (21.1)
	16–30 days	375 (19.25)
	31–60 days	453 (23.25)
	61–91 days	220 (11.29)
	92–182 days	258 (13.24)
	183–365 days	231 (11.86)

*PIM* Potentially Inappropriate Medications, *SD* Standard Deviation

#### Healthcare utilization associated with PIMs use

The number of hospitalizations in non-PIM was 172 (8.8%) versus 346 (17.8%) in PIM users at 1 year follow-up, ED visits were 30 (1.5%) vs. 84 (4.3%), respectively (Table 3). Cox proportional regression model yielded the crude Hazard ratio (cHR) of 2.08 (95% CI: 1.73–2.49) for hospitalization and 2.85 (95% CI: 1.88–4.32) for ED visits. When the model was adjusted by unmatched comorbidities and long-term care facility residing status, the adjusted HR (aHR) presented 2.18 (95% CI: 1.81–2.63) for hospitalization and 3.08 (95% CI: 2.01–4.72) for ED visits (Table 4).

**Table 3:** Risk of healthcare utilization associated with PIM use. A) Distribution of healthcare utilization outcomes in each group

Outcomes	PIM non-user (*n* = 1,948)	PIM user (*n* = 1,948)	*p*-value
Hospitalization*, n (%)	172 (8.83)	346 (17.76)	< 0.01
ED visits*, n (%)	30 (1.54)	84 (4.31)	< 0.01
Hospitalization episode#, mean (sd)	0.11 (0.39)	0.26 (0.65)	< 0.01
ED visit episode, mean (sd)	0.03 (0.34)	0.06 (0.33)	< 0.01
Outpatient episode, mean (sd)	21.42 (18.99)	32.20 (22.73)	< 0.01
Outpatient episode including ED visit, mean (sd)	21.46 (18.99)	32.29 (22.73)	< 0.01

*PIM* Potentially Inappropriate Medications, *CI* Confidence Interval, *ED *Emergency Department

*Number of individuals having at least one hospitalization/ED visit at 1 year

^#^Consecutive hospitalization in the same institution having multiple claims were defined as one episode

**Table 4:** Risk of healthcare utilization associated with PIM use. B) Evaluation of healthcare utilization risk

	Cases and proportion, *n* (%)		Hazard ratio (95% CI)	
	PIM non-ser	PIM user	Crude	Adjusted
Hospitalization*, n (%)	172 (8.8)	346 (17.8)	2.08 (1.73–2.49)	2.18 (1.81–2.63)
ED visit*, n (%)	30 (1.5)	84 (4.3)	2.85 (1.88–4.32)	3.08 (2.01–4.72)

PIM: Potentially Inappropriate Medications, CI: Confidence Interval, ED: Emergency Department

*Number of individuals having at least one hospitalization/ED visit at 1 year

### Associations between healthcare expenditures and PIM use

Healthcare costs presented an increasing trend by the number of PIMs prescribed in all areas of healthcare service domains except for ED visits. Polypharmacy (≥ 5 medications) significantly increased the healthcare costs, specifically using over 7 PIMs prominently contributed to increased costs. The long duration of prescribed PIM also presented increased costs in outpatient visit costs, but the trend was not consistent in other healthcare service areas (Table 5).

**Table 5:** Healthcare expenditure by PIM categories across different healthcare service domains (*n* = 1,948) (Units: US. Dollar*)

*N*		Total	Hospitalization	ED visits#	Outpatient visits
Number of PIM, mean (sd)					
1–2	594	1080.6 (1610.34)	223.94 (1344.65)	8.94 (52.17)	847.72 (657.96)
3–4	640	1407.49 (2270.26)	394.78 (1978.14)	7.63 (48.23)	1005.07 (827.64)
5–6	387	1822.81 (3124.57)	697.48 (2935.5)	8.27 (39.71)	1117.05 (968.02)
7–18	327	3767.46 (7116.89)	2259.1 (7016.69)	23.34 (77.01)	1485.02 (1281.71)
Duration of PIM, mean (sd)					
1–15	411	1156.11 (2824.79)	409.43 (2672.22)	13.8 (62.35)	732.88 (578.65)
16–30	375	1357.93 (3378.21)	448.14 (3200.29)	11.35 (65.37)	898.44 (734.07)
31–60	453	1406.52 (2439.58)	464.36 (2299.4)	7.1 (39.96)	935.07 (780.96)
61–91	220	2290.82 (3828.3)	1036.41 (3499.61)	11.56 (51.2)	1242.85 (978.33)
92–182	258	2242.82 (2699.55)	897.37 (2418.89)	10.37 (49.2)	1335.08 (1084.92)
183–365	231	3358.82 (6754.24)	1680.19 (6680.21)	11.53 (50.4)	1667.11 (1531.15)

*PIM* Potentially Inappropriate Medications, *ED* Emergency Department, *SD* Standard Deviation

*Korean Won is exchanged to US dollar value on 12.31.2018 (1 US. Dollar = 1113.5 Korean Won)

**Fig. 2:**
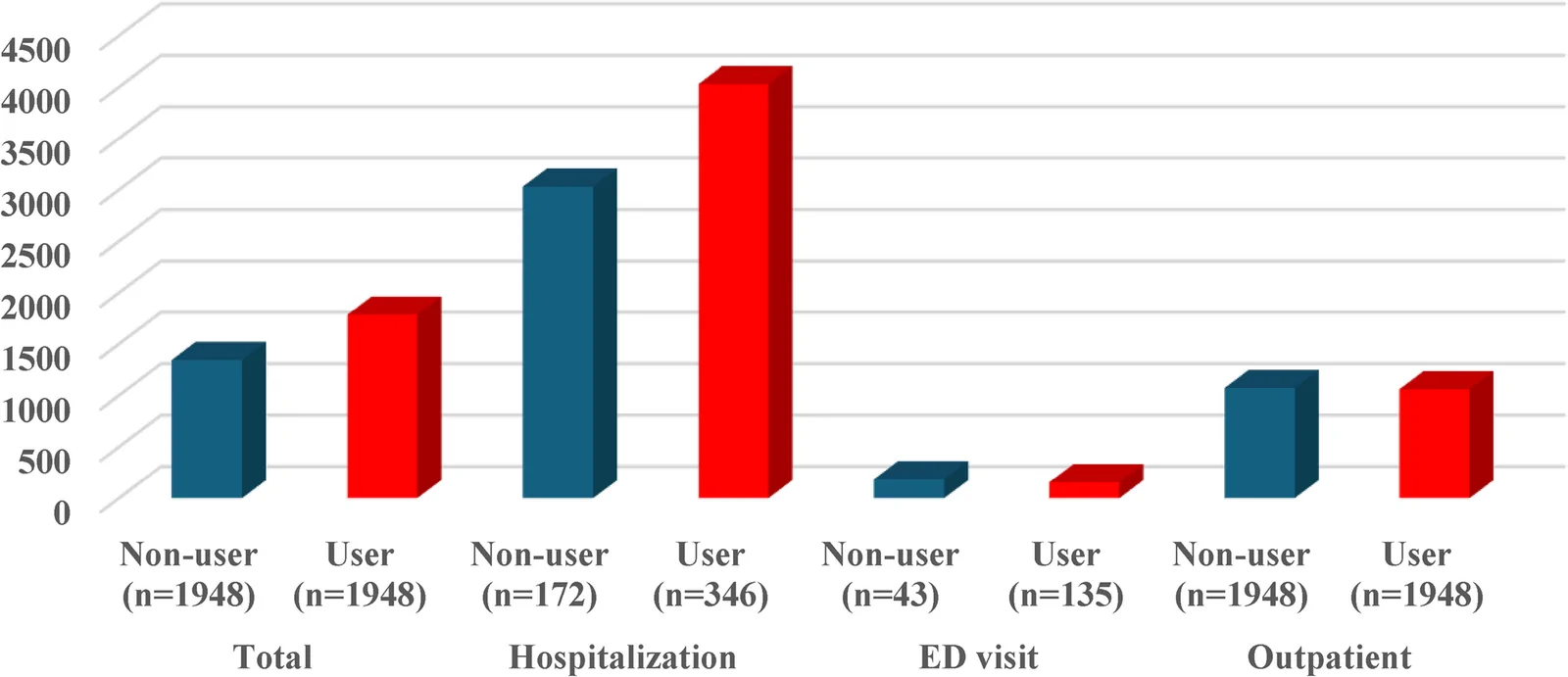
Health care costs associated with PIM use among individuals who had outcomes of interest (second part model)

In the two-part model for the cost analysis, the first model showed significantly increased cost in hospitalization (adjusted OR (aOR): 2.41, 95% CI: 1.97–2.95) and ED visits (aOR: 3.14, 95%: 2.03–4.84) in the PIM-users. Logistic regression was not performed for outpatient cost since all the individuals had outpatient cost (Table S4). In the second part cost analysis, the mean total healthcare costs summing up hospitalization, ED visits, and outpatient costs were higher for PIM users (non-user: $1343.57 (sd: $1993.56) vs. user: $1786.48 (sd: $3708.94)). Hospitalization presented higher costs in PIM users as well. However, ED visits and outpatient costs were not significantly different between the two groups. GLM log link test adjusting for unmatched variables presented 1.44 times (95% CI: 1.35–1.54) higher aCR in total healthcare cost and similarly 1.44 times higher aCR (95% CI: 1.18–1.76) in PIM users (Table 6; Fig. 2).

**Table 6:** Healthcare costs associated with PIM use among individuals who had outcomes and incurred healthcare costs (second part of the two-part model

	PIM non-user	PIM user		Mean Cost Ratio by GLM (95% CI)	
	Mean (sd) in dollar	Mean (sd) in dollar	*P*-value (t-test)	Crude CR	Adjusted CR#
Costs comparison among the individuals who had outcomes					
Total	1343.57 (1993.56)	1786.48 (3708.94)	< 0.01	1.2 (1.12, 1.29)	1.44 (1.35, 1.54)
Hospitalization	3029.89 (4960.65)	4029.88 (7482.68)	0.071	1.33 (1.09, 1.62)	1.44 (1.18, 1.76)
ED visit	184.85 (170.79)	155.75 (140.81)	0.265	0.84 (0.62, 1.14)	1.12 (0.8, 1.59)
Outpatient	1071.96 (940.74)	1059.9 (864.35)	0.677	0.99(0.93, 1.05)	1.06(1, 1.11)

ED: Emergency Department, SD: Standard Deviation, GLM: Generalized Linear Model

*Korean Won is exchanged to US dollar value on 12.31.2018 (1 US. Dollar = 1113.5 Korean Won)

# Variables not included in matching such as hypertension, atrial fibrillation, heart failure, chronic kidney disease, arthritis, and residential status in long-term care facility were adjusted

Significant results made in bold type

## Discussion

In this matched cohort study, we found that PIM prescriptions were highly prevalent in older adults in Korea and associated with increased healthcare utilization and costs. PIM users are associated with a 2.18 times higher hospitalization as well as 3.08 times higher ED visits than PIM non-users at 1 year follow-up. In our previous SCCS analysis using the same database demonstrated PIM exposure period showed increased hospitalization (adjusted incidence rate ratio (aIRR):2.01, 95% CI: 1.97–2.04) and ED visits (aIRR:1.55, 95% CI:1.69–1.79) compared to non-exposure period among the older adults who had outcomes of interest [9]. Despite these methodological differences, both studies consistently demonstrated that PIM use was associated with increased hospitalization and ED utilization. The current study is adding up more generalized findings in the older adults who did not have outcomes of interest at the index date and followed for 1 year for PIM use outcomes in both healthcare and cost areas using matching strategy to minimize confounding in observation study.

Total healthcare utilization related costs were 1.44 times higher for patients who were prescribed PIMs. This is in line with findings of various previous studies regardless of community dwelling or healthcare facility dwelling older adults [19]. In the previous study, the increased healthcare costs in PIM users were attributed to hospitalization associated fees, the increased healthcare service utilization and costs associated with PIM use were mainly occurred in the hospital and rehabilitation settings [20], which was similarly found in our study, hospitalization associated costs were significantly higher in PIM users and the effect size of adjusted CR in total costs and hospitalization is 1.44 implicating hospitalization cost is closely related with total cost. Fick et al. conducted a retrospective study utilizing Medicare managed care data and found that PIM exposure was associated with higher total, provider, and facility related costs [21]. A recent systematic review using 20 articles concluded that PIMs use is associated with higher costs of hospitalization, health care expenses, and ED visits owing to adverse events. This study defined the risk factors associated with adverse PIM effects were highest number of PIMs in use, age older than 75 years, male gender, general health status in older people in use of benzodiazepines, and drug interactions in older people diagnosed as having dementia [22].

In our study, over 36% older adults used more than 5 PIMs in 1 year period, and 11.86% individuals used PIM throughout the year. Healthcare costs increased linearly with the number of PIMs used. A similar trend was observed for hospitalization-, ED visits-, and outpatient visit-related costs. However, the association between prolonged PIM exposure and higher healthcare costs should not be interpreted as causal. Long-term PIM use may reflect poorer underlying health status, greater multimorbidity, frailty, or other healthcare needs that require ongoing treatment, rather than being the sole driver of increased healthcare utilization and costs.

Use of five or more medications simultaneously is defined as polypharmacy. Polypharmacy is particularly prevalent in elderly frail (age > 70 years), it may unnecessarily expose individuals to risk of adverse health outcomes [23]. It has been associated with increased total healthcare expenditure in older adults [24]. The addition of medications to treat an unrecognized adverse reaction can also result in adverse effects and costs increase. Polypharmacy was associated with a higher risk of hospitalization [25, 26] and all-cause death among elderly individuals [27].

The top 10 causes of hospitalization in the older adults were listed by Becker’s hospital review. They are infections such as sepsis, COVID-19, pneumonia, and urinary tract infection, cardio, cerebrovascular events such as heart failure, arrhythmia, heart attack and stroke, muscular skeletal events as osteoarthritis, fracture or fall, and acute or unspecific kidney failure [28].

PIMs are well known for adverse drug effects which increase the risk of hospitalization among older adults. For example, benzodiazepines cause somnolence, lightheadedness, and impaired coordination, thereby significantly increasing the risk of falls and fractures. Cardiovascular medications such as digoxin and amiodarone can cause hypotension or bradycardia, leading to dizziness and syncope. NSAIDs are frequently attributed to severe gastritis and upper GI bleeding. or long-term NSAID use can precipitate acute kidney injury or electrolyte imbalance, and an increased risk of cardiovascular events, particularly with long-term use. Certain antibiotics, such as nitrofurantoin, may also contribute to renal impairment in susceptible older adults. In addition, anticholinergics can cause delirium, cognitive impairment, blurred vision, urinary retention, and gastrointestinal disturbances, all of which may increase the risk of falls, fractures, and hospitalization among older adults [29]. As NSAIDs and anticholinergics were the most frequently prescribed PIMs in our study, their adverse effects may have contributed to the increased risk of hospitalization through mechanisms such as falls, fractures, kidney injury, and cardiovascular events.

Importantly, some of the medications which have been commonly targeted for deprescribing among older adults such as anticholinergics, pain medications including NSAIDs, and proton pump inhibitors (PPIs) still are used widely and prescribed consistently [30]. PIM Pain medication was used the most. Use of more than one pain medication (NSAID) has been shown to increase the risk of gastrointestinal toxicity [31] as well as NSAIDs is one of the medications most commonly associated with adverse drug events in older populations. Of note, usually gastrointestinal drugs such as PPIs follows NSAIDs prescription to prevent NSAIDs associated gastrointestinal adverse effects [32]. A previous study reported that PPIs accounted for the largest proportion of PIM-associated healthcare costs [33], and gastrointestinal drugs including PPIs were the third most frequently used PIMs in this study.

All these results taken together, our findings suggest the importance of regular medication review and optimization of PIM use in older adults. When clinically appropriate, deprescribing may be considered as one component of medication optimization. Deprescribing is the process of supervised medication discontinuation or dose reduction to reduce PIM use or medication errors associated with polypharmacy [34]. Although the present study cannot establish a causal relationship, careful medication optimization, including appropriate deprescribing, may contribute to safer medication use and potentially reduce unnecessary healthcare utilization and expenditure.

Given that NSAIDs and anticholinergics were the most frequently prescribed PIMs in this study, medication optimization strategies may include selecting safer therapeutic alternatives, such as acetaminophen when appropriate, and limiting the duration of NSAID use based on individual clinical conditions and treatment goals. These approaches may help reduce medication-related risks while maintaining adequate symptom control.

If more detailed clinical information from such as Electronic Health Record (EHR) is available when the PIMs were prescribed, we can more accurately determine whether deprescribing or prescription optimization would be appropriate in specific healthcare situations and provide more individualized recommendations. However, these details could not be identified using a secondary claims database. Therefore, this study can only provide general guidance regarding the potential impact of PIM use and the importance of medication optimization among older adults.

Several known contributing factors of PIMs prescribing include lack of communication between different prescribers, healthcare provider’s lack of knowledge in geriatric medicine and pharmacology [35, 36] and the number of prescribers [35]. A recent systematic review and meta-analysis proved that interventions to address PIM prescribing were associated with reductions in the number of medications prescribed, although no substantial other outcome changes were proved [37]. In another systematic review study with 47 articles involving 52 to 124,802 patients, the studies examined various types of interventions such as medication review, educational strategies, clinical decision support system, and organizational and multifaceted approaches. In the hospital, the most successful intervention was medication review (75.0%), while in primary care, educational strategies were the most effective [38]. In the focus groups study with healthcare practitioners, community pharmacists, physicians and nurse practitioners were invited and surveyed for deprescribing strategies in their clinical practice. The top 5 actions highly agreed and prioritized (68%) by participants were as follows; (1) providing patient education, (2) allocating dedicated time and resources for deprescribing within their day, (3) nurturing health care provider collaboration and communication, (4) build a positive provider-patient relationships facilitating patient engagement, and (5) public awareness campaign [39]. These studies really highlight the importance of educational and communicational strategy to increase awareness in healthcare professionals and the public. Patients would like to take fewer medications if they could but often rely on clinicians to take the initiative to start the conversation [40, 41].

This study has several important strengths. First, it extends our previous study beyond the evaluation of healthcare outcomes alone and examining both the healthcare utilization and cost burden associated with PIM use. Among PIM users, total healthcare costs increased almost linearly with the number of PIMs used. Furthermore, costs related to hospitalization, emergency department visits, and outpatient services also increased with increasing numbers of PIMs. These findings are considered a meaningful contribution of this study. Second, we attempted to minimize potential confounding at the index date utilizing a propensity score derived matching rather than simply using a standard regression model. Theoretically, achieving balance in baseline variables minimizes bias at the study index date and improves the validity of subsequent outcome comparisons [42, 43]. When analyzing observational data, propensity score matching is a gold-standard approach to approximate the conditions of a randomized controlled trials by creating comparable groups based on observed covariates [44, 45].

Regarding limitations, first, although we were able to balance the PIM users and non-users with respect to important variables available in the insurance claims data, residual inherent confounding due to unmeasured factors may still exist and could have influenced the observed outcomes. There is no universally accepted approach to adequately account for unmeasured confounding factors and the bias they may introduce [46].

Second, the relationship between PIM use and increased healthcare costs remains uncertain with the possibility of reverse causation. Because PIMs may be repeatedly prescribed throughout the study period, it is difficult to determine whether higher healthcare costs were a consequence of prior PIM exposure before hospitalization or whether PIMs were prescribed during or after hospitalization as part of ongoing clinical management. Furthermore, although long-term use of multiple PIMs may contribute to future adverse events and increased healthcare costs, the relationship may also operate in the opposite direction. Older adults with multimorbidity and frailty may require continued use of medications that are potentially inappropriate to manage complex symptoms, and the associated healthcare costs may reflect the underlying disease burden and treatment needs rather than PIM use alone.

Third, the selection of PIM non-users may have introduced some degree of selection bias after the index date. We defined PIM non-users as individuals with no history of PIM use before or during the 1-year follow-up period, which was intended to identify individuals who remained true non-users throughout the observation period. This approach was adopted because the number of PIM users was substantially higher than that of non-users. However, the strict inclusion and exclusion criteria applied to both groups, along with propensity score matching based on major comorbidities and Charlson Comorbidity Index scores, were used to minimize baseline differences and reduce potential selection bias at the index date.

Fourth, we excluded patients with cancer and those who died during the study period from both groups (PIM users: 7,839/98,892, 7.9%; non-users: 1,032/19,969, 5.2%). This decision was based on the recognition that cancer treatment and end-of-life care are associated with disproportionately high healthcare utilization, including frequent ED visits and hospitalizations, as well as substantially increased end-of-life health care spending [47]. Including these individuals may have introduced outliers in the cost analysis. However, these exclusions may underestimate the healthcare burden associated with PIM use in real-world settings.

Fifth, we assessed co-medication during the 1 month before the index date to capture patients’ medication status immediately prior to cohort entry rather than cumulative historical medication exposure. Our objective was to characterize baseline medication burden at study entry, which we considered the most relevant measure for propensity score matching and adjustment. However, assessing concomitant medications within only 1 month before the index date may not fully capture patients’ overall medication use patterns. In addition, we were unable to verify the use of as-needed (PRN) medications in the claims database. These limitations may have resulted in overestimation or underestimation of actual medication exposure and may have contributed to residual confounding in the matching process.

Additionally, we were unable to account for more granular geographic information and detailed residential characteristics, which may influence healthcare access, healthcare utilization patterns, and financial status. These unmeasured factors may have affected the study outcomes and contributed to residual confounding. Finally, we discussed potential clinical pathways linking PIM use to hospitalization, such as falls, fractures, delirium, gastrointestinal bleeding, and acute kidney injury; however, we were unable to directly evaluate these specific outcomes in the current study. Future studies incorporating more detailed clinical information are needed to investigate the underlying mechanisms of hospitalization associated with PIM use.

## Conclusions

PIMs continue to be prescribed at high rates among older adults in Korea. Our results suggest that PIM use is associated with increased healthcare burdens through higher healthcare utilization and costs. These results emphasize the importance of careful medication optimization, including the implementation of appropriate deprescribing strategies, in the care of older adults. Future research should focus on practical approaches to implementing deprescribing interventions in real-world clinical settings. Furthermore, educational initiatives and improved communication regarding deprescribing strategies among healthcare professionals, caregivers, and older adults may facilitate safer and more cost-effective healthcare delivery.

## Supplementary Information

Supplementary Material 1.

## Data Availability

The data that support the findings of this study are available from NHIS (National Health Insurance Service) of Korea but restrictions apply to the availability of these data, which were used under license for the current study, and so are not publicly available. Data are, however, available from the authors upon reasonable request and with permission of NHIS.
